# Adverse perinatal outcomes and its associated factors among adult and advanced maternal age pregnancy in Northwest Ethiopia

**DOI:** 10.1038/s41598-021-93613-x

**Published:** 2021-07-07

**Authors:** Temesgen Getaneh, Azezu Asres, Toyiba Hiyaru, Selamawit Lake

**Affiliations:** 1 Department of Midwifery, College of Health Science, Debre Markos University, P.O. Box 269, Debre Markos, Ethiopia; 2 Department of Midwifery, College of Health Science, Bahir Dar University, Bahir Dar, Ethiopia

**Keywords:** Paediatric research, Outcomes research, Paediatrics, Public health

## Abstract

Even though reduction of neonatal mortality is needed to achieve Sustainable Development Goals 2030, advanced maternal age is still an independent and a substantial risk factor for different adverse perinatal outcomes, in turn causes neonatal morbidity and mortality. In Ethiopia, research has validated that advanced maternal age is a significant factor in adverse perinatal outcomes, but researches which addressed or estimated its adverse perinatal outcomes are limited, reported inconsistent result and specifically no study was done in the study area. Therefore, this study was aimed to compare adverse perinatal outcomes and its associated factors among women with adult and advanced maternal age pregnancy in Northwest Ethiopia. Comparative cross-sectional study was conducted in Awi Zone, public hospitals, Northwest Ethiopia. Systematic random sampling was employed to select 348 adult and 176 advanced aged pregnant women. Structured questionnaire were used to collect the data. The collected data were analyzed using Statistical Package for the Social Sciences version 25. Binary and multivariate logistic regressions were fitted to assess the association between adverse perinatal outcomes and explanatory variables. P-value less than 0.05 was used to declare statistical significance. Significant percentage of advanced aged women (29.1%) had adverse perinatal outcomes compared to (14.5%) adult aged women. Similarly, proportion low birth weight, preterm birth and low Apgar score were significantly higher among advanced maternal age. The odds of composite adverse perinatal outcomes were higher among advanced maternal age women when compared to adult aged women (AOR 2.01, 95% CI 1.06, 3.79). No formal education (AOR 2.75, 95% CI 1.27, 5.95), short birth interval (AOR 2.25, 95% CI 1.07, 4.73) and complications during pregnancy (AOR 2.12, 95% CI 1.10, 4.10) were also factors significantly associated with adverse perinatal outcomes. Being advanced maternal age is at higher risk for adverse perinatal outcomes compared to adult aged women. Maternal illiteracy, short birth interval and complications during pregnancy were also significantly associated with adverse perinatal outcomes. Access of equal education, provision of family planning and perinatal care (including early detection and management of complication) is recommended.

## Introduction

Advanced maternal age (AMA) pregnancy is considered in a pregnant women who has an estimated delivery date established for a time when a mother is 35 years of age or older^1,2^. Evidence from Canada, low and middle income countries and South Africa showed that 22.6%, 12.3% and 17.5% of AMA pregnancy respectively^3–5^. Postponing marriage until later, the availability of better contraceptive options, social and cultural shifts, wider opportunities for further education and career advancement have impacted AMA prevalence^6,7^. Fertility is reduced as women age, with a significant reduction in ovarian oocyte reserves after the age of 35 years^6,8^.

Even though, one of the major Sustainable Development Goals needed to achieve at 2030 is reduction of neonatal death and improving of their health, neonatal mortality is still unacceptably high^9^. Globally, adverse birth outcomes are the major causes of neonatal morbidity and mortality and represent a gap in the ability to reach Sustainable Development Goal targets^10,11^.

AMA is a significant factor and a major contributor to different adverse perinatal outcomes as compared with adult pregnancy^12–14^. Evidence showed that AMA increased the risk of still birth, intrauterine fetal growth restriction (IUGR), and neonatal death^15–17^. It also associated with other perinatal morbidities including low birth weight (LBW), preterm birth and low Apgar score^17,18^. Similarly, AMA also predisposes a pregnancy to a increased congenital malformation and chromosomal abnormality like trisomy^19^. Study from Denmark showed that adverse neonatal outcomes among AMA women was 10.8% compared to 5.4% among adult aged women^20^.

Recent publications also reported that AMA pregnancy was associated with increased risk of additional neonatal morbidities including large for gestational age, small for gestational age (SGA) and risk of Neonatal Intensive Care Unit (NICU) admission irrespective of parity^21,22^. So, increasing maternal age without a clear cutoff is an independent and substantial risk factor for those adverse outcomes, contribute to the persistent global neonatal mortality^23,24^.

Most of these adverse outcomes are related to the aging process alone, even though coexisting factors such as multiple gestation, higher parity, and chronic medical conditions, are less likely to be observed in younger women^25^. Lack of Antenatal Care (ANC), pre-existing medical diseases along with obstetric complications and poor obstetric history are also associated with neonatal morbidity and mortality^14,26–30^.

Specifically in Ethiopia, Mini Ethiopian Demographic Health Survey in 2019 showed that neonatal mortality was still sustained (29/1000 deaths in 2016 vs 30/1000 deaths in 2019)^31^.

Despite advanced age a major contributor for adverse perinatal outcomes, most Ethiopian studies do not estimate adverse perinatal outcomes of AMA pregnancy. The research focus given to outcomes of advanced age populations specifically in Ethiopia is limited (not done in the study area)^32,33^ and majorly used secondary data. Therefore, this study was aimed to compare adverse perinatal outcomes and its associated factors among women with adult and advanced maternal age pregnancy.

In a country like Ethiopia where striving to reduce neonatal mortality in 2030, investigating such under studied topic will have paramount input for future perinatal health improvement especially in the study area where such research not done. Any gaps in perinatal morbidity and mortality may inform policy makers and program implementers, to pass evidence based informed decisions and target neonatal outcomes.

## Methods

### Study area and period

Institutional based comparative cross sectional study was conducted at Awi zone, Amhara region, Ethiopia. Awi zone is one of the zones found in Amhara Regional State of Ethiopia. Awi Zone is bordered on the West by Benishangul-Gumuz Region, on the North by North Gondar Zone and on the East by West Gojjam. The administrative center of Awi Zone is Injibara^34^. According to the 2007 Central Statistical Agency of Ethiopia report, among 491,077 females live in Awi zone, 232,443 were reproductive age group (15–49), 114,660 were adult women and 58,306 were AMA women^35^. According to the 2018/19 annual report of Awi zone health office, there are five public hospitals and 47 health centers that serve for a total population of 1,077,144^36^. This study was conducted from February 25/2020 to April 25/2020.

### Study population and eligibility criteria

All women with the age of ≥ 20 years old who gave birth at 28 weeks of gestation or greater in Awi zone public hospitals were included in this study. Those women with age range of 20–34 years old (inclusive) were grouped as adult aged women while 35 years old and above were classified as AMA women.

### Sample size and sampling procedure

**Figure 1:**
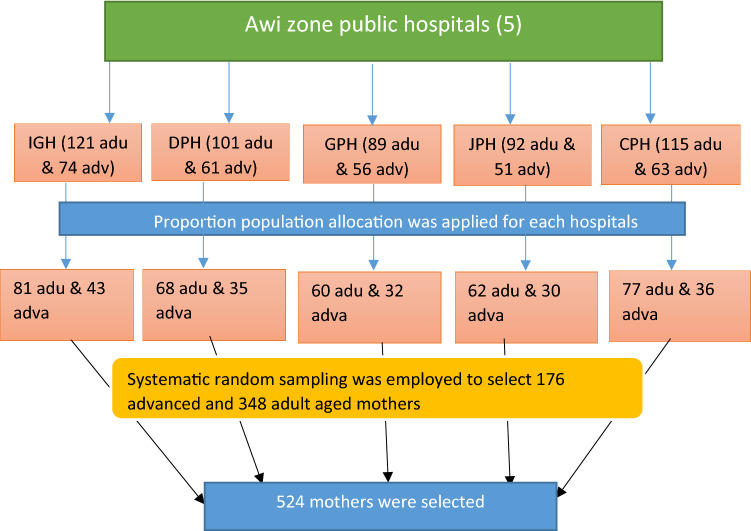
Schematic presentation of sampling procedure to select 524 women, at Awi Zone public hospitals, Ethiopia 2020.

Sample size was calculated using double population formula using Epi-info version 7. Then the following assumptions were considered: 95% two sided level of confidence, a power of 80%, 2 to 1 ratio of adult and advanced aged women and 10% non-response rate. The proportion of different adverse perinatal outcomes among adult and advanced age women pregnancy based on the study conducted in Southern Ethiopia^32^ which resulted maximum sample size was selected. From this study, the proportion of LBW among adult and AMA (3.3% and 10.4% respectively) gave a maximum sample size of 524 mothers (176 advanced age and 348 adult mothers). All five public hospitals found in Awi zone were included in this study. The previous year average delivery report of two months of each hospitals with similar season was used to proportionally allocate the calculated sample size and getting sampling fraction (k-value) (calculated using population size divide by sample size i.e. the calculated k-value was 2, nearly similar for both populations and all public hospitals). The first mother was selected using simple random sampling technique among mothers who gave birth on the day of data collection (then, after the first mother selected, the next was continued based on their discharge from postpartum ward). Finally, systematic random sampling technique was employed to select the final study participants till the required sample size for each facility was saturated (Fig. 1).

### Definition of outcomes

Advanced maternal age: is considered when maternal age is greater or equal to 35 years old^15,37^. Adult maternal age: is considered when maternal age is 20–34 years-inclusive^20,32^. Adverse perinatal outcome (unfavorable): the occurrence of at least one of the following: LBW, preterm birth, low Apgar score at first or fifth minutes after birth, still birth, gross congenital anomaly or neonatal death within 24 h^38,39^. Low Apgar score: is when the neonate delivered with the Apgar score of less than 7 in the first or fifth minute of delivery^40^. Congenital anomaly: is when the newborn has been diagnosed with gross congenital anomaly (hydrocephalus, spinal bifida, anencephaly, cleft lip or pallet and polydactyl)^20^. Bad obstetric history: is considered when the women had at least one of the following condition in previous pregnancy: still birth, early neonatal death and recurrent abortion (three and above spontaneous consecutive abortion)^41^.

### Data collection tool and procedure

Data collection tool was adapted after reviewing different related articles and documents^3,24,32,42–44^. First the questionnaire and checklist was prepared in English version then translated in to Amharic version and then to local language (Agew). Finally, it was translated back to their respective original language versions to check its consistency. All translations were conducted by professional translators in each specific languages. The tool was pretested and reviewed with senior researchers to check reliability and validity. First a discussion meeting from midwifery, public health, nurse department professionals were conducted to review draft questionnaire items. In the meeting, experts provided input on each item's relevance, face validity, and decipherability in the working environment. Items were added (Rhsus status), and the questionnaire wording were modified for clarity and updated for editing error. Otherwise the questionnaire was consistent during asking. An interviewer administered and chart reviewing, structured questionnaire was used to collect the data. The tool contain a total of four major parts (mother’s sociodemographic data, obstetric related data, life style, chronic medical disease related data and neonatal outcomes) and 54 questions. Six diploma Midwives and senior BSC midwife were recruited as data collectors and supervisor respectively. One-day training was given for all data collectors and supervisor by principal investigators about the objective of the study, study tool, data collection method, procedure and how to fill the questionnaires. All pregnant women who gave birth at Awi Zone public hospitals were interviewed and their chart was reviewed after admitted to labor and delivery ward after informed written consent was obtained. All methods were performed in accordance with the relevant guidelines and regulations.

### Data quality assurance

One-day training was given for data collectors and supervisor on the aim of study, data collection tool and procedures. The tool was pretested at on Dure Bete primary hospital 5% of the sample size to ensure consistency and completeness of questioners. Data collectors were supervised throughout the course of data collection period. Then, the overall process was coordinated and controlled by principal investigator. Principal investigators, supervisor and data collectors were taken a discussion meeting daily after the end of data collection to ensure completeness. Furthermore, the collected data were entered in to Epi-data (with double entry) computer programs version 3.1 to minimize data entry error.

### Data processing and analysis

The collected data were entered using Epi data version 3.1 computer program. Then, it was exported to Statistical Package for the Social Sciences (IBM SPSS) version 25 for analysis. Descriptive statistics like frequency and summary statistics were employed to describe characteristics of the study population. Chi square and independent t-test were used to compare categorical and continuous variables between adult and advanced maternal age women. Binary and multivariate logistic regression analysis were conducted to identify factors associated with adverse perinatal outcomes. All explanatory variables in binary logistic regression with p-value 0.25 or less were considered for multivariate logistic regression analysis to control confounding factors.

Adjusted Odds Ratio (AOR) with their corresponding 95% Confidence Intervals (CI) was used to declare the presence of association between dependent and independent variables. *p*-value less than 0.05 was used to declare statistical significance in this study.

### Ethics approval and consent to participate

Ethical clearance was obtained from Institutional Review Board of Bahir Dar University’s College of Medicine and Health Sciences (study protocol number-0058/2020). Then, officials at different levels in the hospitals were communicated through letters from College of Medicine and Health Science. Then, informed written consent was obtained from all participants. For women who cannot read and write/illiterate the informed consent was obtained from the legally authorized representative. Confidentiality of the information was secured throughout the study process. Finally, the results of study was used only for study purpose.

### Consent for publication

Not applicable.

## Results

### Socio-demographic characteristics

In this study, a total of 520 participants were included giving a response rate of 99.2%. The mean age ± standard deviation (SD) of adult and advanced aged mothers was 25.8 (± 3.1) and 37.5 (± 2.8) years respectively. More than two-third 241 (69.9%) of adult mothers were urban resident compared with 117 (66.9%) of AMA women were rural resident. In regarding to educational status, more than half 108 (61.7%) of AMA women had no formal education compared to 63 (18.3%) adult aged women. All populations (both advanced and adult aged mothers) were non-alcohol user and non-smoker (Table 1).

**Table 1:** Socio-demographic and life style characteristics of mothers who gave birth in Awi Zone Public Hospitals, Ethiopia, 2020.

Variables	Advanced age (175)	Adult age (n = 345)	Total (n = 520)
	Frequency (%)	Frequency (%)	Frequency (%)
**Residence**			
Urban	58 (33.1%)	241 (69.9%)	299 (57.5%)
Rural	117 (66.9%)	104 (30.1%)	221 (42.5%)
**Marital status**			
Single/separated	3 (1.7%)	8 (2.3%)	11 (2.1%)
Married /union	171 (97.7%)	335 (97.1%)	506 (97.3%)
Others^a^	1 (0.6%)	2 (0.6%)	3 (0.6%)
**Maternal education**			
Not read and write	108 (61.7%)	63 (18.3%)	171 (32.9%)
Primary	35 (20%)	124 (35.9%)	159 (30.6%)
Secondary and above	32 (18.3%)	158 (45.8%)	190 (36.5%)
**Ethnicity**			
Amhara	175 (100%)	343 (99.4%)	518 (99.6%)
Others^b^	0	2 (0.6%)	2 (0.4%)
**Religion**			
Orthodox	173 (98.9%)	335 (97.1%)	508 (97.7%)
Others^c^	2 (1.2%)	10 (2.9%)	12 (2.3%)
**Maternal occupation**			
House wife	64 (36.6%)	162 (47%)	226 (43.5%)
Farmer	86 (49.1%)	62 (18%)	148 (28.5%)
Government employ	16 (9.1%)	66 (19.1%)	82 (15.8%)
Others^d^	9 (5.1%)	55 (15.9%)	64 (12.3%)
**Husband occupation**			
Farmer	116 (66.3%)	87 (25.2%)	203 (39%)
Government employ	32 (18.3%)	99 (28.7%)	131 (25.3%)
Merchant	15 (8.6%)	101 (29.3%)	116 (22.3%)
Others^e^	12 (6.9)	58 (16.8%)	70 (13.5%)
**Income**			
Mean ± Standard deviation	2910.29 ± 288.045	4612.64 ± 453.756	4166.85 ± 734.28

^a^Divorced and widowed.

^b^Oromo and Benishangul Gumz.

^c^Muslim and protestant.

^d^Student, merchant and private employ.

^e^Private employ and driver.

### Obstetrics characteristics

Twenty four (14%) AMA women had short birth interval, similar with adult aged women 24 (15.2%). Nearly 35% (60) of AMA women had previous bad obstetrical history, compared with 16 (10.1%) adult aged women. More than one-third 35.4% (62) of AMA women had unplanned pregnancy compared with 8.7% (30) of adult aged women. One hundred seventy one (97.7%) AMA and 339 (98.3%) adult aged women had ANC follow up. But, only 57 (33.3%) AMA women were initiate ANC at 12 weeks or before compared to 184 (54.3%) adult aged women. In contrast, there was no significant differences between AMA and adult women regarding tetanus toxoid vaccination (92.6% vs 94.5%) and iron folate supplementation (95.4% vs 92.2%) respectively (Table 2).

**Table 2:** Obstetrics characteristics of mothers who gave birth in Awi Zone Public Hospitals, Ethiopia, 12020.

Variables	Advanced age (175)	Adult age (n = 345)	Total (n = 520)
	Frequency (%)	Frequency (%)	Frequency (%)
**Middle upper arm circumference**			
≥ 23 cm	158 (90.3%)	319 (92.5%)	477 (91.7%)
< 23 cm	17 (9.7%)	26 (7.5%)	43 (8.3%)
**Rh status**			
Positive	160 (91.4%)	320 (92.8%)	480 (92.3%)
Negative	15 (8.6%)	25 (7.2%)	40 (7.7%)
**Birth interval**			
< 24 months	24 (14%)	24 (15.2%)	48 (14.5%)
≥ 24 months	148 (86%)	134 (84.8%)	282 (85.5%)
**Previous bad obstetrical history**			
Yes	60 (34.9%)	16 (10.1%)	76 (23%)
No	112 (65.1)	143 (89.9%)	225 (77%)
**No. of pregnancy**			
Singleton	165 (94.3%)	339 (98.3%)	504 (96.9%)
Twin	10 (5.7%)	6 (1.7%)	16 (3.1%)
**Status of pregnancy**			
Planned	113 (64.6%)	315 (91.3%)	428 (82.3%)
Unplanned	62 (35.4%)	30 (8.7%)	92 (17.7%)
**ANC follow up**			
Yes	171 (97.7%)	339 (98.3%)	510 (98.1%)
No	4 (2.3%)	6 (1.7%)	10 (1.9%)
**Number of visit**			
1–3 visit	101 (59.1%)	132 (38.9)	233 (45.7)
≥ 4 visit	70 (40.9)	207 (61.1)	277 (54.3)
**Gestational age when start ANC**			
≤ 12 weeks	57 (33.3)	184 (54.3)	241 (47.3)
> 12 weeks	114 (66.7)	155 (45.7)	269 (52.7)
**Tetanus toxoid vaccine**			
Yes	162 (92.6%)	326 (94.5%)	488 (93.8%)
No	13 (7.4%)	19 (5.5%)	32 (6.2%)
**No of Tetanus toxoid vaccine**			
Ones	40 (24.7)	34 (10.4)	74(15.2)
≥ two times	122 (75.3)	292 (89.6)	414 (84.8)
**Iron folate supplementation**			
Yes	167 (95.4%)	319 (92.5%)	486 (93.5%)
No	8 (4.6%)	26 (7.5%)	34 (6.5%)
**Iron folate months**			
< 3 months	52 (31.1)	69 (21.7)	121 (24.9)
≥ 3 months	115 (68.9)	249 (78.3)	364 75.1)
**Male partner involvement**			
Yes	96 (54.9%)	195 (56.5%)	291 (56%)
No	79 (45.1%)	150 (43.5%)	229 (44%)
**Gravidity**			
Primigravida	3 (1.7%)	187 (54.2%)	190 (36.5%)
2–5	98 (56%)	157 (45.5%)	255 (49)
Grand muligravida	74 (42.3%)	1 (0.3%)	75 (14.4%)
**Parity**			
Nulliparous	3 (1.7%)	1.79 ± 0.095	198 (37.7%)
2–5	117 (66.9%	193 (55.9%)	266 (51.2%)
Grand multipara	55 (31.4%)	149 (43.2%)	58 (11.2%)
**Gestational age at delivery**			
Mean ± standard deviation	38.39 ± 0.15	39.03 ± 0.083	38.92 ± 1.73
**Onset of labor**			
Spontaneous	133 (76.4%)	294 (85.7%)	427 (82.6%)
Induced	41 (23.6%)	49 (14.3%)	90 (17.4%)
**Mode of delivery**			
Spontaneous vaginal	128 (73.1%)	199 (57.7%)	327 (62.9%)
Cesarean section	19 (10.9%)	31 (9%)	50 (9.6%)
Instrumental vaginal delivery	28 (16%)	115 (33.3%)	143 (27.5%)
**Fetal presentation**			
Vertex	166 (94.9%)	329 (95.4%)	495 (95.2%)
Others^a^	9 (5.1%)	16 (4.6%)	25 (4.8%)
**Duration of labor**			
≤ 12 h	156 (90.7%)	263 (76.9%)	419 (81.5%)
> 12 h	16 (9.3%)	79 (23.1%)	95 (18.5%)

^a^Breech, shoulder and face.

### Obstetric and medical complications characteristics

Around 21% (37) of AMA women had complication during pregnancy, compared with 14.5% (50) of adult aged women. Complications during labor-delivery were significantly more common among AMA women 41 (23.4%) than adult aged women 55 (15.9%) (Table 3).

**Table 3:** Obstetrics and medical characteristics of mothers who gave birth in Awi Zone Public Hospitals, Ethiopia, 2020.

Variables	Advanced age (n = 175)	Adult age (n = 345)	Total (n = 520)
	Frequency (%)	Frequency (%)	Frequency (%)
**Complication during pregnancy**			
Yes	37 (21.1%)	50 (14.5%)	87 (16.7%)
No	138 (78.9%)	295 (85.5%)	433 (83.3%)
**Complication during labor-delivery**			
Yes	40 (22.9%)	56 (16.2%)	96 (18.5%)
No	135 (77.1%)	289 (83.8%)	424 (81.5%)
**Type of complication during pregnancy and labor-delivery**			
Pregnancy induced hypertension	18 (10.28%)	25 (7.24%)	43 (8.26%)
Premature membrane rapture	12 (6.8%)	20 (5.79%)	32 (6.15%)
Prolonged labor	13 (7.42%)	24 (6.95%)	37 (7.11%)
**Chronic medical illness**			
Yes	34 (19.4%)	23 (6.7%)	57 (11%)
No	141 (80.6%)	322 (93.3)	463 (89%)

### Perinatal characteristics

Independent t-test showed that the mean (± SD) birth weight between newborns of AMA and adult mothers was statistically different (3005.71 (± 44.89) vs 3118.26 (± 27.21)) gram respectively. Of identified causes of perinatal death, prematurity and asphyxia accounts 9.1% and 36.4% respectively. Significant percent of newborn born from AMA women 33 (18.9%) had low first minute Apgar score compared to 36 (10.4%) adult aged women newborns(Table 4).

**Table 4:** Perinatal characteristics of mothers who gave birth in Awi Zone Public Hospitals, Ethiopia, 2020.

Variables	Advanced age (n = 175)	Adult age (n = 345)	Total (n = 520)
	Frequency (%)	Frequency (%)	Frequency (%)
**Sex of the newborn**			
Male	98 (56%)	192(55.7%)	290 (55.8%)
Female	77 (44%)	153 (44.3%)	230 (44.2%)
**Outcome of the newborn**			
Alive	169 (96.6%)	340 (98.6%)	509 (97.9%)
Dead	6 (3.4%)	5 (1.4%)	11 (2.1%)
**Type of death**			
Still birth	3 (50%)	4 (66.7%)	3 (50)
Immediate neonatal death	3 (50%)	1 (37.3%)	3 (50)
**Cause of death**			
Prematurity	0	1 (20%)	1 (9.1%)
Asphyxia	2 (33.3%)	2 (40%)	4 (36.4%)
Unknown cause	4 (66.7%)	2 (40%)	6 (54.5%)
**Weight in gram**			
Mean ± SD	3005.71 ± 44.89	3118.26 ± 27.21	3081.15 ± 537.2
**Weight for gestational age of the newborn**			
SGA	12 (6.9%)	15 (4.3%)	27 (5.2%)
Appropriate for gestation age	156 (89.1%)	314 (91%)	470 (90.4%)
Large for gestational age	7 (4%)	16 (4.6%)	23 (4.4%)
**First minute Apgar**			
< 7	33 (18.9%)	36 (10.4%)	69 (13.3%)
≥ 7	142 (81.1%)	309 (89.6%)	457 (86.7%)
**Fifth minute Apgar**			
< 7	8 (4.6%)	9 (2.6%)	17 (3.3%)
≥ 7	167 (95.4%)	336 (97.4%)	503 (96.7%)
**NICU admission**			
Yes	36 (20.6%)	46 (13.3%)	82 (15.8%)
No	139 (79.4%)	299 (86.7%)	438 (84.2%)
**Neonatal outcomes**			
Un-favorable	51 (29.1%)	50 (14.5%)	101 (19.4%)
Favorable	124 (70.9%)	295 (85.5%)	419 (80.6%)

### Adverse perinatal outcomes

**Figure 2:**
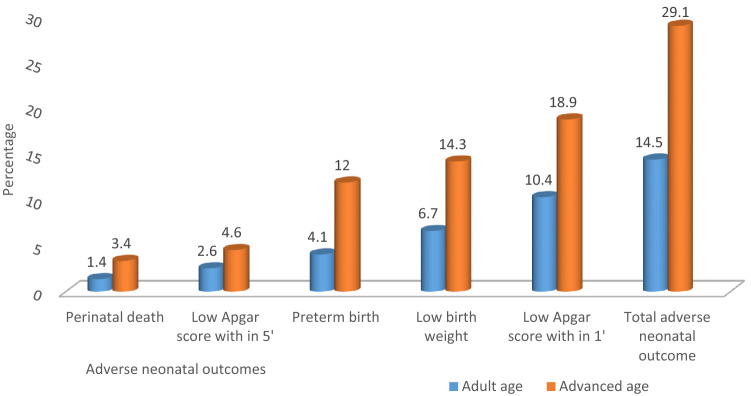
Adverse perinatal outcomes among adult and advanced age mothers who gave birth at Awi zone public hospitals, Ethiopia, 2020.

Prevalence of adverse perinatal outcome was 29.1% (95% CI 22.9, 36) and 14.5% (95% CI 10.7, 18.3) among advanced and adult aged maternal respectively. Overall, prevalence of adverse perinatal outcome was 19.4% (95% CI 15.8, 22.9). The proportion of preterm birth among AMA women (12%) was significantly higher compared to (4.1%) adult aged women (p < 0.001). similarly, significant percentage of babies born from AMA women were LBW compared to adult aged newborn babies (14.3% vs 6.7% respectevily with p < 0.001) (Fig. 2).

### Factors associated with adverse perinatal outcomes

Binary logistic regression was employed to evaluate association between different sociodemographic, obstetric and medical related variables with adverse perinatal outcomes. The variables including maternal age, residence, maternal educational status, ANC follow up, tetanus toxoid vaccination, iron folate supplementation, birth interval, previous bad obstetrical history, complication during recent pregnancy and labor-delivery which had p-value ≤ 0.25 in binary logistic regression were further analyzed with multivariate logistic regression.

Model fitness was tested with Hosmer and Lemeshow Goodness of Fit test (p = 0.86). In addition, there was no problem of interaction effect between variable and collinearity among independent variables with standard error of < 0.2.

After controlling all factors listed below in the regression table, the odds of adverse perinatal outcomes among advanced aged women were 2.01 times higher when compared with adult aged women (AOR 2.01, 95% CI 1.06, 3.79). The likelihood of adverse perinatal outcomes among women who had no formal education were 2.75 times higher when compared with women who had secondary and above educational level (AOR 2.75, 95% CI 1.27, 5.95). In addition, women who had short birth interval (< 24 months) were 2.25 times more likely to have adverse perinatal outcomes when compared with their counterparts (AOR 2.25, 95% CI 1.07, 4.73). Moreover, the odds of adverse perinatal outcomes among women who had complication during pregnancy were 2.12 times higher when compared with their counterparts (AOR 2.12, 95% CI 1.10, 4.10) (Table 5).

**Table 5:** Logistic regression to identify factors associated with adverse perinatal outcomes among adult age and advanced age mothers who gave birth in Awi Zone public hospitals, Ethiopia, 2020.

Variables	Adverse perinatal outcomes				
	Frequency (%)		COR (95% CI)	AOR (95% CI)	p-value
	Yes	No			
**Maternal age**					
Advanced (> 34)	51 (29.1)	124 (70.9)	2.42 (1.55, 3.77)	2.01 (1.06, 3.79)	0.030
Adult (20–34)	50 (14.5)	295 (85.5)	1	1	
**Residence**					
Urban	42 (14)	257 (86)	1	1	0.724
Rural	59 (26.7)	162 (73.3)	2.22 (1.43, 3.46)	0.87 (0.40, 1.87)	
**Maternal educational status**					
Not read and write	51 (29.8)	120 (70.2)	2.95 (1.66, 5.23)	2.75 (1.27, 5.95)	0.010
Primary	20 (12.6)	139 (87.4)	2.26 (1.36, 3.77)	2.01 (0.96, 4.20)	0.063
Secondary and above	30 (15.8)	160 (84.2)	1	1	
**Bad obstetric history**					
Yes	24 (31.6)	52 (68.4)	2.04 (1.14, 3.64)	1.27 (0.65, 2.46)	0.476
No	47 (18.4)	208 (81.6)	1	1	
**ANC follow up**					
Yes	94 (18.4)	416 (81.6)	1	1	0.736
No	7 (70)	3 (30)	10.3 (2.6, 40.6)	1.47 (0.15–14.15)	
**Iron folate**					
Yes	90 (18.5)	396 (81.5)	1	1	0.354
No	11 (32.4)	23 (67.6)	2.1 (0.9, 4.4)	1.89 (0.49–7.32)	
**Tetanus Toxiod vaccine**					
Yes	87 (17.8)	401 (82.2)	1	1	0.462
No	14 (43.8)	18 (56.2)	3.5 (1.7, 7.4)	1.55 (0.48–5.01)	
**Birth interval**					
< 24 months	15 (31.3)	33 (68.8)	1.83 (0.93, 3.60)	2.25 (1.07, 4.73)	0.031
≥ 24 months	56 (19.9)	226 (80.1)	1	1	
**Complication during pregnancy**					
Yes	29 (33.3)	58 (66.7)	2.5 (1.50, 4.18)	2.12 (1.10, 4.10)	0.025
No	72 (16.6)	361 (83.4)	1	1	
**Complication during labor and delivery**					
Yes	29 (30.2)	67 (69.8)	2.11 (1.27, 3.50)	1.85 (0.94, 3.64)	0.073
No	72 (17)	352 (83)	1	1	

## Discussion

The overall prevalence of adverse perinatal outcome was 19.4% (95% CI 15.8, 22.9). This figure is in agreement with studies conducted in South Nation and Nationality of People^14^, Gondar^11^ and Tigray^45^. This could implicated that adverse perinatal outcome is still a public health threat and interventions such as advising pregnancy at adult level and especial perinatal care for those population are important. In addition, this study finding is higher than finding of study done in Kembata Tembaro Zone^46^. Nearly 95% of study participants of study done in Kembata Tembaro Zone were adults^46^. Pregnancy of adult aged women is less likely to have adverse perinatal outcomes. However, this result is lower than findings of studies done in Hosanna^47^, North Wollo Zone^48^ and Dessie^39^. In all these studies^39,47,48^, adolescent women were included as study participants which increased risk of adverse perinatal outcomes^49^. Consistently, this finding is also lower than result of similar study done in Jima^32^. This could be due to currently there is improvement of family planning and perinatal care service provision when compared to the service given in 2016 (as the study was done in this year)^31^. The difference might be related to variation in socio-cultural, study setting and period.

The percentage of adverse perinatal outcome among AMA women was significantly higher compared with adult aged women. This finding is consistent with result of studies held in Denmark^44^, Japan^20^, Australia^50^ and Jima^32^. This is due to the evidence that AMA is associated with a range of obstetrical complications and medical comorbidities which in turn predispose to different adverse perinatal outcomes^43,51^.

In regarding to specific adverse perinatal outcomes, significant percentage of AMA women (12%) had preterm birth when compared to (4.1%) adult aged women. It is supported with results of studies conducted in Turkey^42^, United Kingdom^43^, Italy^10^, Finland^52^ and Tigray^33^. This is due to the reason that the risk of developing medical and obstetrical complications could be increased when age of the mothers advances^17,53^. These co-morbidities may also associated with increased risk of early labor induction or pregnancy termination^54^. Similarly, higher proportion of AMA women (14.3%) had LBW when compared to (6.7%) adult aged women. This result is consistent with studies done in Australia^53^, a meta-analysis in Portugal^55^, Taiwan^56^ and South Africa^5^. This may be due to the evidence that AMA is associated with increased risk of co-morbidities such as chronic hypertension, pregnancy induced hypertension and placenta abruption, which in turn causes placental insufficiency, preterm labor, IUGR, SGA and other poor obstetric outcomes^10,25,37^.

Furthermore, this study also showed that babies born among AMA women had lower first minute Apgar score compared babies born from adult women. This figure is in track with studies done in South Korea^23^, Sweden^24^ and meta-analysis done in Portugal^55^. It is evidenced that, AMA is at increased risk of medical and obstetric complications. So that, perinatal morbidity like prematurity, poor fetal growth and LBW are more common in AMA women which increased risk of birth asphyxia or low Apgar score^17,57^.

Maternal age was significantly associated with adverse perinatal outcomes. It was found that AMA women were more likely to have adverse perinatal outcome compared to adult women. This result is in line with studies conducted in Developing countries birth registry^28^, Sweden^24^, United Kingdom^51^, Poland^21^, Hawassa^14^ and Debre Tabor^12^. The possible explanation could be the fact that AMA is one of the non-modifiable risk factors for different adverse neonatal outcomes including preterm birth, LBW and still birth. In addition, it could be related to ageing process alone or increased risk of coexisting factors such as multiple gestation and chronic medical conditions^17,57^ as well as obstetrical complications including preeclampsia, preterm labor, placental abruption and IUGR)^3,22^.

Concerning to maternal education, the odds of adverse neonatal outcome among women who had no formal education was higher when compared with women who had secondary and above educational level. This result is in line with findings of studies held in United States of America^58^, China^22^, Debre Tabor^12^ and North Wollo Zone^59^. This is could be due to the relationship between non-eduacted with low resource income which leads to traditional dietary practice and low decision power to access good quality of maternal health services^60^. In addition, uneducated women could be unaware to attend ANC and institutional delivery, less likely to carry out essential newborn care (like breast feeding) and dietary behavioral modifications^61,62^.

According to the present study, women who had short birth interval (< 24 months) was associated with adverse perinatal outcome. This is supported with findings of studies done in Bangladesh^63^, Afghanistan^29^, Turkey^64^, Egypt^65^ and Tigray^66^. This could be due to the fact that short birth interval is associated with inadequate iron folate and other nutrient replenishment which (leads to anemia and nutrient depletion)^67,68^. In addition, short birth interval could not allow sufficient time to recover from the stress of the previous pregnancy, associated with cervical insufficiency and placental abruption that increases multiple adverse perinatal outcomes like LBW, IUGR, preterm birth, SGA and perinatal death^63,69,70^.

Complication during pregnancy was also significantly associated with adverse perinatal outcomes. This finding is consistent with studies done in China^71^, Bangladesh^72^, Uganda^73^, Nigeria^74^, Kenya^75^, Gurage Zone^47^, Gondar^11^, Tigray^76^ and Jima^27^ and systematic review done in Ethiopia^77^. This could be due to most complications are associated with decreased placental nutrient, which results placental insufficiency)^78^. It is evidenced that different obstetric complications were associated with LBW, preterm birth and perinatal death^79^. In addition, some life threatening pregnancy complications may be lead to obstetric interventions regardless of gestational age^80^. Generally, this study finding will be used as a baseline evidence specfiaclly in the study area where no previous study was conducted. In addition, it will be applied as guide for health professionals and advanced maternal age mothers in regarding to pregnancy outcomes of advanced maternal age pregnancy.

Finally, this study shares the limitation of cross sectional study that may not indicate causal relationship. In the two comparision group, there may be difference in physiological and hormonal conditions in adding to age difference; confounder for the association. In addition, as the study was done in hospital setting, perinatal outcome of women who gave birth at home was not assessed. Finally, our study misses adverse perinatal outcomes after 24 h of birth.

## Conclusion

Generally, one out of five neonates develop adverse perinatal outcomes. Thus, older age remains an independent risk factors doe adverse outcome. In adding this, substantial proportion of preterm birth, LBW and low first minute Apgar score were seen among AMA women when compared to adult women. No formal eduaction, short birth interval and complications during pregnancy were factors significantly associated with adverse perinatal outcomes.

Therefore, Ethiopian Ministry of Health with its stake holders should focus on advocating equal access of education and promotion for all women, provision of family planning and perinatal care (including early detection and management of complication) for all reproductive age women to optimize their health and to have favorable neonatal outcomes.

## Data Availability

The datasets analyzed during the current study are available from the corresponding author upon reasonable request.

## Abbreviations

AMA: Advanced maternal age
ANC: Antenatal care
IUGR: Intra-uterine growth restriction
LBW: Low birth weight
NICU: Neonatal intensive care unit
SGA: Small for gestational age
SPSS: Statistical package for the social sciences
